# ACE2‐ and HR2‐Mimetic Peptides Inhibit Replication of Two SARS‐CoV‐2 Variants

**DOI:** 10.1002/jmv.71060

**Published:** 2026-07-20

**Authors:** Melvin E. Zúñiga‐Hernández, Karen L. Reyes‐Barrera, Sergio N. Hidalgo‐Figueroa, Edgar D. Páez‐Pérez, Clara I. Espitia Pinzón, Ángel G. Alpuche‐Solís

**Affiliations:** ^1^ Laboratorio de Biología Molecular de Plantas, División de Biología Molecular Instituto Potosino de Investigación Científica y Tecnológica, A.C. San Luis Potosí SLP México; ^2^ Departamento de Inmunología, Instituto de Investigaciones Biomédicas Universidad Nacional Autónoma de México (UNAM) Ciudad de México México; ^3^ División de Biología Molecular, Instituto Potosino de Investigación Científica y Tecnológica A.C., Secretaría de Ciencia, Humanidades Tecnología e Innovación San Luis Potosí SLP México; ^4^ Geomicrobiología, Metalurgia Universidad Autónoma de San Luis Potosí San Luis Potosí SLP México

**Keywords:** ACE2‐mimetic peptides, binding assay, blocking entry, HR2‐mimetic peptides, microneutralization, SARS‐CoV‐2 variants

## Abstract

Several therapeutic approaches to inhibiting severe acute respiratory syndrome coronavirus 2 (SARS‐CoV‐2) focus on blocking the virus's entry mechanism. This can be blocked in two ways: by interacting with the receptor‐binding domain (RBD) of the spike protein and human angiotensin‐converting enzyme 2 (ACE2), and by blocking viral‐cellular membrane fusion. Here, we designed mimetic peptides based on ACE2 and the region of the viral spike stem. Through in silico molecular docking, we select the peptides with the highest binding energy. The peptides Nat (P‐Nat) and 3 (P‐3) inhibited RBD–ACE2 interaction in a surrogate ELISA assay by 54% and 58.77%, respectively. On the other hand, P‐Nat and P‐3 inhibited SARS‐CoV‐2 in VeroE6 cells, showing 50% inhibitory concentration (IC_50_) values of 6.28 and 12.79 μM against Wuhan and 6.26 and 7.27 μM against Omicron, respectively. Meanwhile, the stem mimetic peptide (P‐K2) showed an IC_50_ of 29.17 μM against Omicron. Therefore, P‐Nat and P‐3 are good candidates to inhibit both variants of SARS‐CoV‐2, while P‐K2 shows inhibitory activity against the Omicron variant. We propose that these peptides could be used as prophylactic agents prior to viral infection, as they are specifically designed to block viral entry into host cells.

## 1 Introduction

The severe acute respiratory syndrome coronavirus (SARS‐CoV‐2) causes both acute and chronic infections, similar to SARS‐CoV and MERS‐CoV, leading to symptoms such as fever, cough, headache, and pneumonia, among others [1]. To date, SARS‐CoV‐2 has infected over 777 million people and caused more than 7 million deaths worldwide [2]. The secondary attack rate (SAR) refers to the proportion of individuals who become infected after exposure to an index case—the first identified case of a disease within a specific setting. Different SARs have been reported for SARS‐CoV‐2 variants, with rates of 42.7% for Omicron, 36.4% for Alpha, 29.7% for Delta, and 22.5% for Beta [3]. The transmissibility rate has significantly increased, particularly with Omicron, as this variant has evolved the ability to evade both natural and vaccine‐induced immune responses. Mutations in the Spike protein have enabled Omicron to reduce the neutralizing effect of serum antibodies from infected or vaccinated individuals [4, 5, 6, 7].

Vaccination‐acquired immunity helps reduce the progression to severe COVID‐19; however, it does not completely prevent disease transmission. Additionally, individuals with comorbidities such as immunosuppression, cardiovascular disease, diabetes mellitus, chronic kidney disease, cancer, pulmonary disease, and obesity are at a higher risk of developing severe COVID‐19 cases [8, 9]. To mitigate severe outcomes, therapeutic strategies that block viral entry into host cells or inhibit SARS‐CoV‐2 replication are essential. Among these approaches, researchers have explored chemically synthesized compounds, monoclonal antibodies, and, more recently, antiviral peptides as potential treatments.

Paxlovid, a drug composed of nirmatrelvir and ritonavir, functions as a SARS‐CoV‐2 protease inhibitor. It appears to be particularly effective in older adults and significantly reduces the risk of severe COVID‐19 outcomes [10]. Remdesivir is the most widely used antiviral drug against SARS‐CoV‐2. It is an RNA‐dependent RNA polymerase (RdRp) inhibitor that acts within 3 days in high‐risk, nonhospitalized patients [11, 12]. However, its use is limited due to the need for intravenous administration [13, 14]. On the other hand, molnupiravir, an orally administered antiviral, has shown effectiveness in treating COVID‐19 when initiated within 5 days of symptom onset in nonhospitalized, vaccinated, and unvaccinated adults [15, 16, 17]. In contrast, monoclonal antibodies face the same administration challenge as remdesivir and have also proven ineffective against the Omicron variant due to mutations in the Spike protein [18, 19, 20]. The first step in SARS‐CoV‐2 infection is the recognition of the Spike (S) protein by host cell receptors. The S protein consists of two subunits: S1 and S2. The S1 subunit, exposed on the viral surface, contains the receptor‐binding domain (RBD), which specifically recognizes the angiotensin‐converting enzyme 2 (ACE2) on host cells [21]. Following this, the S2 subunit, which contains heptad repeat (HR) regions and a fusion peptide (FP), interacts with the cell surface serine protease TMPRSS2, leading to proteolytic cleavage of the S protein. This cleavage triggers fusion, either through endocytosis or direct binding of the viral and cellular membranes, depending on the availability of cellular proteases [22, 23].

During membrane fusion between viruses and cells, the S protein undergoes a series of sequential conformational changes. First, TMPRSS2 cleaves the S1 subunit, separating it from S2, leaving S2 as the remaining stem. Next, the HR1 and HR2 domains of the S2 subunit begin to unfold: HR1 anchors to the host cell membrane via the FP, while HR2 remains attached to the viral membrane through the transmembrane (TM) region. Once S2 is anchored to both membranes, they gradually draw closer and fuse, enabling viral entry [24]. In response to this mechanism, our research group designed peptides targeting the RBD–ACE2 and HR1–HR2 interaction regions. These peptides aim to block viral binding to the host cell and prevent viral membrane fusion with the host cell membrane. Thus, our work focuses on the design and evaluation of antiviral peptides for inhibiting multiple SARS‐CoV‐2 variants.

## 2 Material and Methods

### 2.1 Design and Three‐Dimensional Modeling of Antiviral Peptides

Two ACE2‐mimetic peptides targeting the RBD of SARS‐CoV‐2, peptide Nat (P‐Nat) and peptide 3 (P‐3) (Table 1), and one peptide derived from the S2 subunit of the Spike protein stem, peptide K2 (P‐K2) (Table 2), were designed. The three‐dimensional structures of the peptides were predicted using the Robetta server with the RoseTTAFold algorithm. The highest‐scoring model was selected, downloaded in PDB format, and used for molecular docking analyses. For peptide modeling against the S2 subunit, Chimera version 1.17.3 was employed. The design was based on HR2‐HR1 core from the post‐fusion crystal structure of the S2 subunit of SARS‐CoV‐2 (PDB: 6M1V). Mutagenesis was performed on the HR2 chain, followed by energy minimization. The final optimized structure was downloaded in PDB format and used for molecular docking analyses.

**Table 1:** Two peptides inhibit the ACE2–RBD interaction. Two segments of the ACE2 protein were used to design the peptides P‐Nat and P‐3.

Name	Target	Sequence
ACE2	RBD	EEQAKTFLDKFNHEAEDLFYQSS—LGKGDFR
P‐Nat	RBD	EEQAKTFLDKFNHEAEDLFYQSSGLGKGDFR
P‐3	RBD	EEEAKIKLDKFNHEAEDLFYQSSLGLGKGDFR

Abbreviations: ACE2, angiotensin‐converting enzyme 2; P‐3, peptide 3; P‐Nat, peptide Nat; RBD, receptor‐binding domain.

**Table 2:** One peptide inhibits membrane fusion. One segment of the HR1 protein was used to design the P‐K2 peptide.

Name	Target	Sequence
HR2	HR1	QISGINASVVNIQKEIDRLNEVAKNLNESLIDLQEL
P‐K2	HR1	GDISGINASEVNIQKEIDRLNEVIKNLNESLIDLQEL

Abbreviations: HR, heptad repeat; P‐K2, peptide K2.

### 2.2 Molecular Docking

Free molecular docking of ACE2‐mimetic peptides to their target protein, the RBD domain of Spike (S) protein, was performed using ICM software. The ACE2–RBD complex crystal structures of the Wuhan strain (PDB ID: 6M0J) and the Omicron variant (PDB ID: 7WPB) were used for this analysis. For the Spike stem‐derived peptide (HR2 subunit), free molecular docking was conducted between the peptide and its target protein, S2. The post‐fusion core crystal structure of the S2 subunit of SARS‐CoV‐2 (PDB ID: 6M1V) was used, as sequence analysis revealed no differences between the Wuhan and Omicron variants in this region. Following docking, the interactions between the peptides and their respective receptors were analyzed, and the −kcal/mol values were obtained, indicating binding affinity to the target protein. The peptides with the highest affinity were selected for synthesis and sent to GenScript for production.

### 2.3 Inhibition Analysis of RBD–ACE2 Binding by Antiviral Peptides

P‐Nat and P‐3 were dissolved in 3% ammonium hydroxide, while P‐K2 was dissolved in 0.045% ammonium hydroxide. The RayBio COVID‐19 Spike‐ACE2 binding assay kit was used to assess the interaction between the RBD protein and ACE2. This kit, which employs a 96‐well plate coated with recombinantly expressed S‐RBD, allowed us to evaluate the inhibitory capacity of the designed peptides against the ACE2–RBD binding region. P‐Nat and P‐3 samples were twofold serially diluted from 100 to 2.5 μM; the diluted peptides were added to the wells, which were incubated for 90 min at room temperature. After incubation, the wells were washed, and the 1X ACE2 protein included in the kit was added, followed by another 90‐min incubation. Subsequently, three washes were performed to remove unbound ACE2, and a 1X anti‐ACE2 antibody was added and incubated for 1 h. After additional washes to eliminate excess antibody, a horseradish peroxidase (HRP)‐conjugated anti‐IgG secondary antibody was introduced. ELISA detection was performed by adding 3,3′,5,5′‐tetramethylbenzidine (TMB) substrate. After a 30‐min incubation, the HRP–TMB reaction was stopped, and absorbance was measured at 450 nm using a Bio‐Rad model 550 microplate reader.

### 2.4 Cell Culture and Viral Propagation

Vero E6 cells were cultured in Dulbecco modified Eagle medium (DMEM) supplemented with 10% fetal bovine serum (FBS) at 37°C in a humidified incubator with 5% CO_2_. The SARS‐CoV‐2 WT D614G and Omicron BA.5.1 variants were used for viral propagation. Viral stocks were prepared by infecting Vero E6 monolayers in DMEM supplemented with 2% FBS. The viral supernatant was harvested 3 days postinoculation and subsequently titrated using the 50% tissue culture infective dose (TCID_50_) assay. Viral propagation was carried out at Biosafety Level III, as required for the experiments.

### 2.5 Cell Viability Assay

The cell viability assay was conducted using Vero E6 cells to evaluate the effects of P‐Nat, P‐3, and P‐K2. Vero E6 cells were seeded into a 96‐well transparent flat‐bottom tissue culture plate containing DMEM supplemented with 2% FBS and incubated for 24 h prior to the assay. Peptide samples were twofold serially diluted starting from 100 to 0.78 μM and incubated with the cells for 96 h at 37°C with 5% CO_2_. The antiviral peptide's ability to prevent cytopathic effect (CPE) was assessed using a neutral red uptake assay, with absorbance measured at 540 nm [25, 26]. The absorbance data were converted into percent cell viability using the following formula:

$$\%\mathrm{Viability}=\frac{\mathrm{Abs}\;\mathrm{sample}-\mathrm{abs}\;\mathrm{not}\;\mathrm{cells}}{\mathrm{Ab}\;\mathrm{smock}-\mathrm{abs}\;\mathrm{not}\;\mathrm{cells}}\times 100\%.$$

### 2.6 CPE‐Based Viral Microneutralization (CPE) Assay for Antiviral Peptides

Vero E6 cells were seeded into a 96‐well cell culture plate in DMEM supplemented with 5% FBS and incubated for 24 h before infection. Peptide samples were twofold serially diluted starting from 100 to 0.78 μM; dilutions of peptides were mixed with an equal volume of viral solution containing 0.02 TCID_50_ of SARS‐CoV‐2 per cell. The peptide‐virus mixture was incubated for 1 h at 37°C with 5% CO_2_ before transferring the mixture to a Vero E6 monolayer at > 90% confluence. The plates were incubated for 96 h at 37°C with 5% CO_2_. CPEs caused by viral replication were assessed using a neutral red uptake assay, with absorbance measured at 540 nm [25, 26]. The absorbance data were transformed into percentage inhibition using the following formula:

$$\%\mathrm{Inhibition}=\frac{\mathrm{Abs}\;\mathrm{sample}-\mathrm{abs}\;\mathrm{mock}}{\mathrm{Abs}\;\mathrm{vehicle}-\mathrm{abs}\;\mathrm{mock}}\times 100\%.$$

### 2.7 Statistical Analysis

GraphPad Prism 6 software was used to perform a one‐way analysis of variance with Dunnett test multiple comparisons, using a significance level (*α*) of 0.05, and was used to generate graphical representations and calculate the 50% inhibitory concentration (IC_50_) and the 50% cytotoxic concentration (CC_50_).

## 3 Results

### 3.1 Molecular Docking

**Figure 1:**
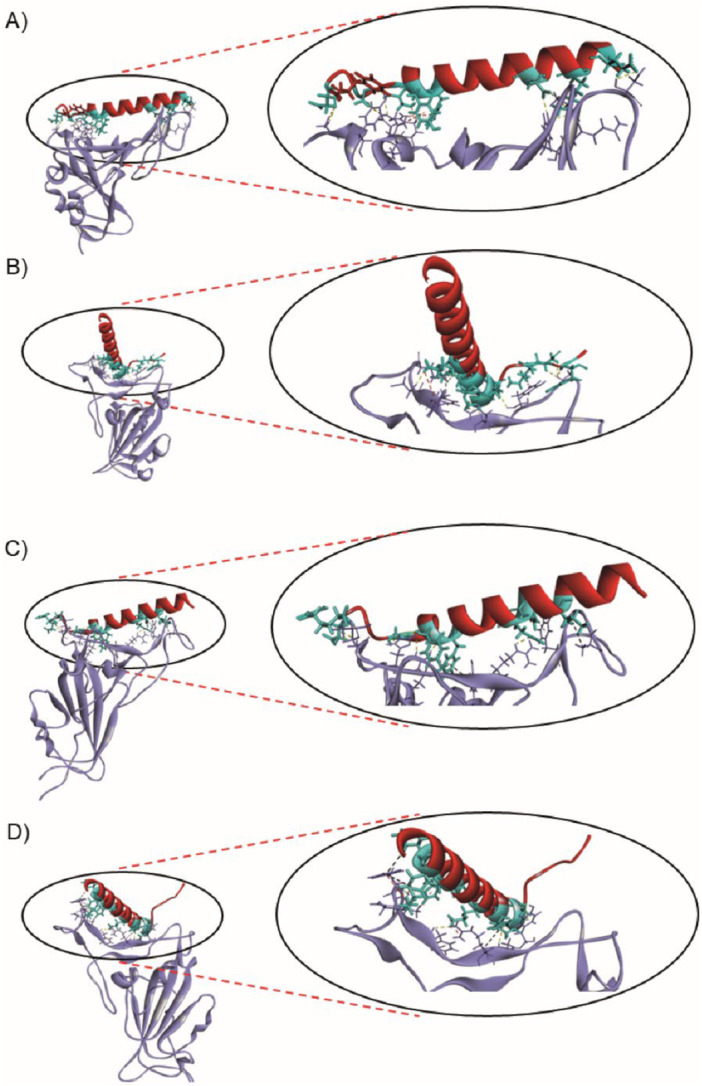
Molecular docking of ACE2‐mimetic peptides with RBD from different SARS‐CoV‐2 variants. P‐Nat (A) and P‐3 (B) were docked with the 6M0J crystal structure of the Wuhan variant. P‐Nat (C) and P‐3 (D) were docked with the 7WPB crystal structure of the Omicron variant. Lilac, RBD protein; red, peptide not interacting with RBD; blue, amino acids interacting with the RBD protein. Bond types: orange, electrostatic; yellow, hydrogen bonds; black, hydrophobic. ACE2, angiotensin‐converting enzyme 2; P‐3, peptide 3; P‐Nat, peptide Nat; RBD, receptor‐binding domain; SARS‐CoV‐2, severe acute respiratory syndrome coronavirus 2.

**Figure 2:**
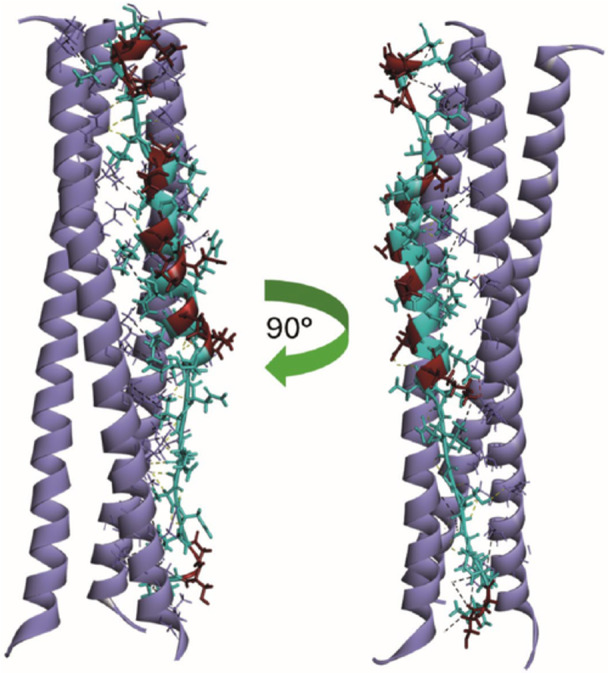
Molecular docking of P‐K2 with HR1 from the 6M1V crystal structure of the Wuhan variant. Lilac, HR1 trimer; red, P‐K2 not interacting with HR1; cyan, peptides interacting with HR1. Bond types: orange, electrostatic; yellow, hydrogen bonds; black, hydrophobic. HR, heptad repeat; P‐K2, peptide K2.

Molecular docking between the ACE2‐ and HR2‐mimetic peptide models (ligands) and the RBD and HR1 domains (receptors) of the Spike protein is crucial for understanding their intermolecular interactions. This approach helps select pharmacologically relevant molecules by identifying key binding interactions. In this study, multiple molecular docking simulations were performed to analyze the types of interactions between each ligand and its respective receptor (Figures 1 and 2).

Therefore, we first designed the ligand models. The ACE2‐mimetic peptide models were predicted using the Robetta server. These peptides were designed to interact with the RBD protein and were designated as P‐Nat (Figure S1A,B) and P‐3 (Figure S1C,D). P‐Nat was created from two regions of the ACE2 protein that interact directly with the RBD protein; these regions are linked by a glycine residue that induces a beta‐turn. P‐3 shares structural similarity with P‐Nat but incorporates specific amino acid modifications to enhance its affinity for the RBD. These modifications involve replacing the amino acids Q, T, and F with E, I, and K, respectively. The objective was to increase the hydrophobic interactions in P‐3.

Additionally, P‐K2 (Figure S1E), which mimics a segment of the HR2 domain of the spike protein, was generated through mutagenesis of the HR2 chain from the 6M1V crystal structure. The amino acids replaced in the HR2 chain were Q, V, and A with G, D, E, and I, increase the hydrophobic interactions. P‐K2 was specifically designed to bind to the HR1 domain of the S2 subunit of the spike protein, potentially interfering with viral membrane fusion.

For the P‐Nat with the Wuhan RBD, 14 interactions were observed, primarily hydrogen bonds, along with a few electrostatic interactions, yielding a binding energy of −50.26 kcal/mol (Figure 1A and Table S1). Similarly, P‐3 with the Wuhan RBD exhibited 14 interactions, including hydrogen bonds, hydrophobic, and electrostatic interactions, with a binding energy of −46.33 kcal/mol (Figure 1B and Table S2). Molecular docking of P‐Nat with the Omicron RBD revealed 12 interactions, comprising hydrogen bonds, hydrophobic, and electrostatic interactions, yielding a binding energy of −47.76 kcal/mol (Figure 1C and Table S3). In contrast, P‐3 with the Omicron RBD formed 13 interactions, also involving hydrogen bonds, hydrophobic, and electrostatic interactions, with a binding energy of −49.14 kcal/mol (Figure 1D and Table S4).

Finally, P‐K2 docking to the Wuhan HR1 trimer yielded 50 interactions, primarily hydrogen bonds and hydrophobic interactions, resulting in a binding energy of −92.43 kcal/mol (Figure 2 and Table S5). These findings highlight the potential of the designed peptides to bind their target regions effectively, supporting their use as antiviral candidates against SARS‐CoV‐2.

### 3.2 Analysis of the Inhibition of RBD–ACE2 Protein Binding by Antiviral Peptides

**Figure 3:**
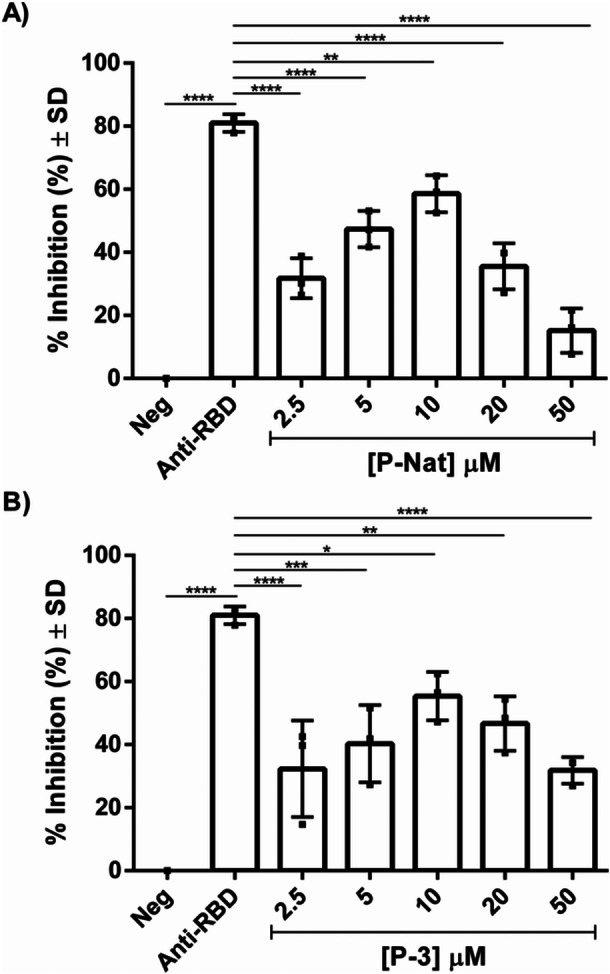
Quantification of the inhibition percentage of ACE2–RBD binding for P‐Nat (A) and P‐3 (B). Statistically significant differences are indicated by asterisks. One‐way ANOVA with Dunnett test multiple comparisons were used (*p < 0.05; **p < 0.01; ***p < 0.001; ****p < 0.0001). Anti‐RBD: monoclonal IgG antibody purified in mammalian cells. ACE2, angiotensin‐converting enzyme 2; ANOVA, analysis of variance; IgG, immunoglobulin G; P‐3, peptide 3; P‐Nat, peptide Nat; RBD, receptor‐binding domain.

The inhibition percentage of the RBD–ACE2 interaction was quantified using a surrogate ELISA assay. P‐Nat and P‐3 were evaluated at concentrations ranging from 2.5 to 50 μM, exhibiting similar inhibition patterns. The highest inhibition of RBD–ACE2 binding was observed at 10 μM, with P‐Nat achieving 58.77% inhibition and P‐3 reaching 54% (Figure 3A,B). Notably, P‐Nat also showed inhibition of approximately 50% at 5 μM. However, at concentrations above 10 μM, the inhibition percentage for both peptides declined, possibly due to solubility limitations.

### 3.3 Evaluation of the Inhibition Percentage of SARS‐CoV‐2 Virus Variants (Wuhan D614G and Omicron BA.5.1) Using Synthetic Antiviral Peptides

A viral neutralization assay was conducted against the SARS‐CoV‐2 variants Wuhan D614G and Omicron BA.5.1, using peptide concentrations ranging from 0.78 to 100 μM.

**Figure 4:**
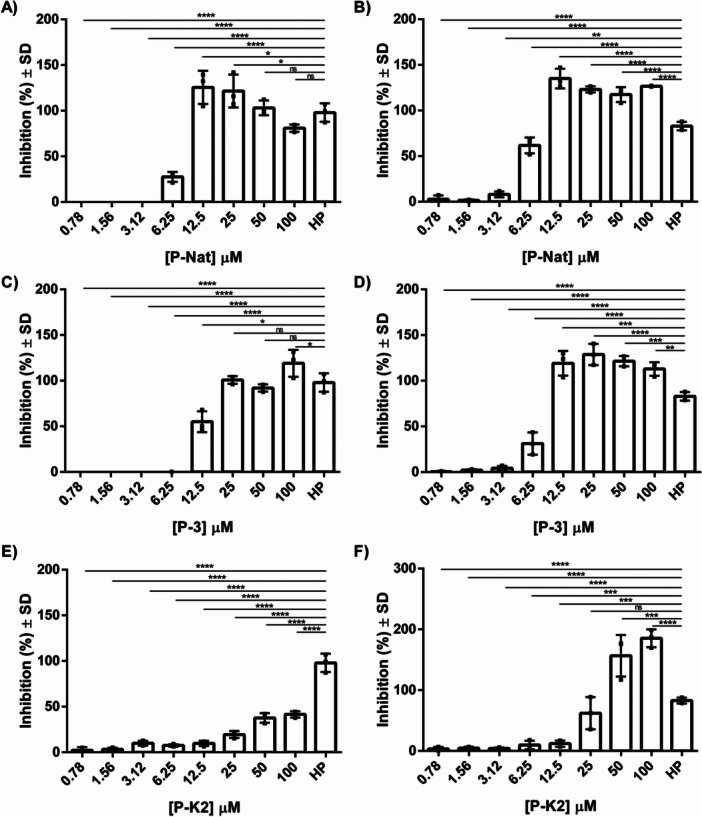
Inhibition percentage of P‐Nat, P‐3, and P‐K2 on the replication of the SARS‐CoV‐2 virus. Wuhan D614G (A, C, E) and Omicron BA.5.1 (B, D, F) variants were used. The effect of different concentrations (ranging from 0.78 to 100 μM) is shown. Statistically significant differences are indicated by asterisks. One‐way ANOVA with Dunnett test multiple comparisons were used (*p < 0.05; **p < 0.01; ***p < 0.001; ****p < 0.0001). HP = human plasma from a vaccinated individual. ANOVA, analysis of variance; P‐3, peptide 3; P‐K2, peptide K2; P‐Nat, peptide Nat; SARS‐CoV‐2, severe acute respiratory syndrome coronavirus 2.

For the Wuhan D614G variant, P‐Nat achieved 100% inhibition at 12.5, 25, and 50 μM, but inhibition decreased to 80% at 100 μM (Figure 4A). Against the Omicron BA.5.1 variant, P‐Nat exhibited 100% inhibition across all tested concentrations from 12.5 to 100 μM (Figure 4B). These results suggest that P‐Nat is highly effective against both SARS‐CoV‐2 variants.

In the viral neutralization assay with P‐3, different inhibition patterns were observed against the Wuhan D614G variant. P‐3 at 25 and 100 μM exhibited 100% inhibition, while at 12.5 and 50 μM, the inhibition was 59% and 91%, respectively (Figure 4C). For the Omicron BA.5.1 variant, P‐3 demonstrated 100% inhibition at concentrations from 12.5 to 100 μM, similar to P‐Nat (Figure 4D).

P‐K2 exhibited distinct inhibition profiles compared with P‐Nat and P‐3. Against the Wuhan D614G, P‐K2 displayed lower inhibition percentages of 37% and 41% at concentrations of 50 and 100 μM, respectively (Figure 4E). In contrast, for the Omicron BA.5.1 variant, P‐K2 achieved 100% inhibition at concentrations of 50 and 100 μM (Figure 4F). These results contrast with P‐Nat and P‐3, both of which inhibited 100% of Omicron BA.5.1 at all tested concentrations (12.5–100 μM).

### 3.4 Cell Viability Assay Using Antiviral Peptides

Cell viability assays were performed for P‐Nat, P‐3, and P‐K2 across concentrations ranging from 0.78 to 100 μM. P‐Nat had no significant effect on cell viability at concentrations ranging from 0.78 to 12.5 μM, with viability remaining close to 100%. However, at higher concentrations, a decrease in viability was observed, with values dropping to 81% at 25 μM, 54% at 50 μM, and 0% at 100 μM, indicating cytotoxicity at 50 and 100 μM (Figure S2A). Similarly, P‐3 followed a comparable trend, maintaining cell viability near 100% between 0.78 and 25 μM. However, at 50 and 100 μM, a decline in viability was observed, suggesting cytotoxic effects at these concentrations (Figure S2B). In contrast, P‐K2 did not affect cell viability across the entire tested concentration range (0.78–100 μM), indicating that it is noncytotoxic at these concentrations (Figure S2C).

### 3.5 Dose‐Response Curves of Antiviral Peptides

The IC_50_ and CC_50_ curves were generated to determine the peptide concentration required to inhibit 50% of viral activity in viral inhibition assays and to induce 50% cell death in cell viability assays. Additionally, the selectivity index (SI) was calculated by comparing each compound's antiviral activity with its cytotoxicity.

**Figure 5:**
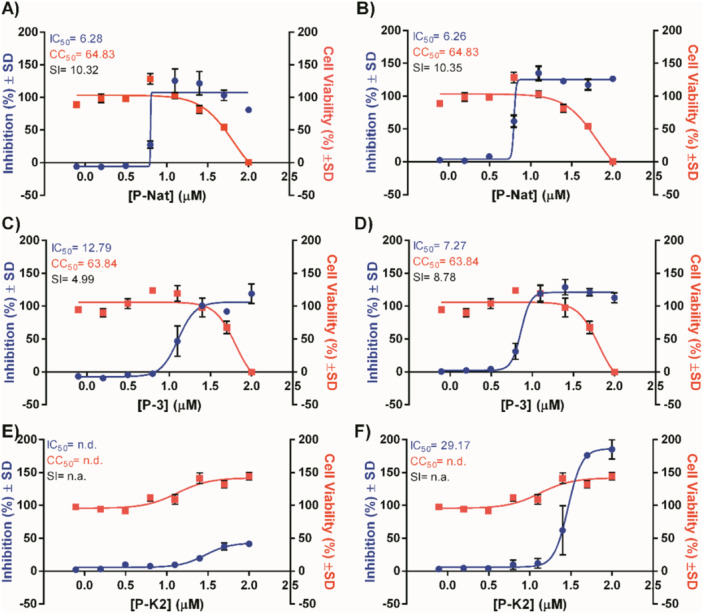
Inhibitory activity and cytotoxicity of P‐Nat, P‐3, and P‐K2 against SARS‐CoV‐2. Wuhan D614G (A, C, and E) and Omicron BA.5.1 (B, D, and F) variants were used. The effect of different concentrations (ranging from 0.78 to 100 μM) is shown. SARS‐CoV‐2 infection of Vero E6 cells at 0.02 TCID_50_. CC_50_, 50% cytotoxic concentration; IC_50_, 50% inhibitory concentration; P‐3, peptide 3; P‐K2, peptide K2; P‐Nat, peptide Nat; SARS‐CoV‐2, severe acute respiratory syndrome coronavirus 2; SI, selectivity index; TCID_50_, 50% tissue culture infective dose.

P‐Nat exhibited an IC_50_ of 6.28 μM against the Wuhan D614G variant (Figure 5A), indicating that a low concentration is required for viral inhibition. Similarly, for the Omicron BA.5.1 variant, the IC_50_ was 6.26 μM (Figure 5B), demonstrating comparable efficacy against both variants. Additionally, P‐Nat had a CC_50_ of 64.83 μM (Figure 5A,B), with SIs of 10.32 and 10.35 for the Wuhan and Omicron variants, respectively. These findings suggest that P‐Nat is equally effective against both viral variants (Figure 5A,B).

P‐3 displayed an IC_50_ of 12.79 μM against the Wuhan D614G variant (Figure 5C), indicating a relatively low concentration is needed for 50% viral inhibition. Against the Omicron BA.5.1 variant, the IC_50_ was 7.27 μM (Figure 5D), which is lower than that for the Wuhan variant, suggesting greater efficacy against Omicron. Additionally, P‐3 had a CC_50_ of 63.84 μM (Figure 5C,D), with an SI of 4.99 and 8.78 for the Wuhan and Omicron variants, respectively. These results indicate that P‐3 is more effective against Omicron than Wuhan (Figure 5C,D).

The IC_50_ for P‐K2 was not determined against the Wuhan D614G variant (Figure 5E) because it did not exhibit inhibitory activity above 50% but showed an IC_50_ of 29.17 μM against the Omicron BA.5.1 variant (Figure 5F). The CC_50_ for P‐K2 was not determined (Figure 5E,F), because it did not exhibit cytotoxicity at any tested concentration. The SI was not determined because P‐K2 was not cytotoxic, indicating it can be used up to 100 μM.

## 4 Discussion

Mimetic peptides are designed to replicate a protein's function while preserving its biological activity. This makes them a valuable strategy for blocking protein‐protein interactions, such as the binding of the SARS‐CoV‐2 Spike RBD to human ACE2. In this study, we mimicked human ACE2 and the HR1 domain of the SARS‐CoV‐2 Spike stem. Analysis of the RBD–ACE2 complex from the 6M0J crystal structure revealed that 17 RBD residues interact with 20 ACE2 residues at distances below 4 Å [27]. In our molecular docking analysis, P‐Nat formed 14 interactions, where eight RBD residues interacted with nine P‐Nat residues (Table S1). Similarly, P‐3 formed 14 interactions, involving nine RBD residues and eight P‐3 residues (Table S2). Notably, in both peptides analyzed in this study and in previous reports, most RBD–ACE2 complex interactions were hydrogen bonds. Furthermore, an analysis of the 7WPB crystal structure for the Omicron RBD–ACE2 complex revealed that Omicron RBD has twice the affinity for ACE2 compared with WT RBD in its monomeric form, and 6–9 times higher affinity in its trimeric form. This increased affinity may be attributed to mutations in the Omicron RBD interaction region, which led to two additional hydrogen bonds and additional interactions between RBD monomers. These changes compensate for the loss of polar interactions between WT RBD and ACE2, while also maintaining the open conformation of the Spike trimer, stabilizing its binding to ACE2 [28]. In our molecular docking analysis of mimetic peptides binding to ACE2 with the Omicron RBD, we observed distinct interaction patterns compared with those with the Wuhan RBD. P‐Nat replaced two hydrogen bonds with one hydrophobic and one electrostatic interaction, while P‐3 replaced five hydrogen bonds with three hydrophobic, one electrostatic, and one noncovalent interaction. These findings suggest that mutations in the Omicron RBD alter interactions with P‐Nat and P‐3 compared with those observed with the Wuhan RBD.

We demonstrated that ACE2‐mimetic peptides effectively inhibited the ACE2–RBD protein‐protein interaction, a crucial mechanism by which SARS‐CoV‐2 enters the host cell. Subsequently, the viral microneutralization assay enabled us to quantify the extent to which these peptides inhibited viral replication. Our results showed that ACE2‐mimetic peptides completely inhibited the Wuhan D614G variant, with P‐Nat being the most effective. At 12.5 μM, P‐Nat achieved 100% viral inhibition, whereas P‐3 required 25 μM to reach the same effect. As a result, P‐Nat exhibited an IC_50_ that was half that of P‐3, which may be attributed to its lower binding energy with RBD (−3.93 kcal/mol), as indicated by molecular docking analysis. This difference is likely due to P‐Nat replacing a C‐H interaction with a hydrophobic one (Tables S1 and S2). Even greater inhibitory effects were observed against the Omicron variant, where both ACE2‐mimetic peptides fully inhibited viral replication at 12.5 μM. In this case, the binding energy difference between the peptides and their receptor was smaller (−1.38 kcal/mol). Although the peptides interacted differently with Omicron RBD, their overall interaction energies were more comparable (Tables S3 and S4). Han et al. (2006) found that six chemically synthesized ACE2‐derived peptides were tested for anti‐SARS‐CoV activity using a pseudovirus model. Two peptides, corresponding to ACE2 residues 22–57 and 22–44, exhibited antiviral activity with IC_50_ values of 6 and 50 μM, respectively. However, a third peptide, composed of two ACE2 regions (residues 22–44 and 351–357), exhibited significantly higher antiviral activity with an IC_50_ of just 0.1 μM [29]. In another study, Karoyan et al. [30], tested ACE2‐mimetic peptides against SARS‐CoV‐2 in Calu‐3 cells, obtaining IC_50_ values of 42, 46, and 53 nM. When comparing our ACE2‐mimetic peptides with those from the previous study, we found that their IC_50_ values fell within a similar range. Specifically, P‐Nat and P‐3 exhibited IC_50_ values of 6.28 and 12.79 μM, respectively, against the Wuhan D614G variant (Figure 5A,C). These results demonstrate that ACE2‐mimetic peptides establish a strong interaction with the RBD of the Spike protein, further supporting their potential as effective viral inhibitors.

On the other hand, P‐K2 showed a lower inhibition percentage than the peptides designed to compete with ACE2. Specifically, P‐K2 did not achieve more than 50% inhibition even at 100 μM, making it less suitable for inhibiting the replication of the Wuhan D614G variant. However, against the Omicron BA.5.1 variant, P‐K2 completely inhibited viral replication at concentrations of 50 and 100 μM, suggesting that P‐K2, which mimics HR2, has an IC50 four times higher than the ACE2‐mimetic peptides. Molecular docking analysis of P‐K2 with its receptor revealed 50 interactions, including hydrogen bonds, hydrophobic interactions, and electrostatic interactions. Compared with the native HR1–HR2 core, P‐K2 exhibited more hydrogen bonds and hydrophobic interactions than the HR2 domain in the 6M1V crystal structure [31]. Additionally, Sun et al. (2006), show that two assays were conducted to assess the inhibition mechanism of an HR2‐mimetic peptide. In a pseudovirus model using Huh7 cells, the peptide exhibited an EC_50_ of 0.32 μM, whereas in a viral inhibition assay using HCoV‐19 in Vero E6 cells, the same peptide showed an EC_50_ of 0.58 μM [31]. In another study, Zhu et al. [32] tested different HR2‐mimetic peptides against pseudoviruses, with IPB02 showing the best activity, an IC_50_ of 0.08 μM. On the other hand, Xia et al. [33] tested peptide targeting HR1 of SARS‐CoV‐2, obtaining an IC_50_ values of 36.5 nM.

These findings suggest that S2‐derived peptides inhibit viral entry by interfering with the conformational transition of the HR1/HR2 complex during membrane fusion.

Cell viability assays conducted with the antiviral peptides produced distinct results. P‐Nat had no impact on cell viability at concentrations ranging from 0.78 to 12.5 μM, maintaining viability levels close to 100%, which was sufficient to completely inhibit viral replication in both tested variants. P‐3 exhibited similar behavior, maintaining cell viability near 100% at concentrations between 0.78 and 25 μM, with 25 μM sufficient to fully inhibit the virus in both strains. P‐K2 showed no cytotoxic effects at any tested concentration, indicating that a 100 μM concentration can be safely used to achieve 100% viral inhibition. These results indicate that the peptides can be used at concentrations that are not cytotoxic and that inhibit 100% of the virus, with optimal concentrations of 12.5 μM for the Nat peptide, 25 μM for P‐3, and 50 μM for the K2 peptide. Finally, we believe that combining Nat or P‐3 peptides with the K2 peptide could have a synergistic effect on viral replication while also reducing the cytotoxicity of ACE2‐mimetic peptides. This must be verified experimentally in a future study.

## 5 Conclusion

The P‐Nat, P‐3, and P‐K2 are promising candidates for inhibiting SARS‐CoV‐2 replication, effectively targeting both the Wuhan D614G and Omicron BA.5.1 variants. Among them, P‐Nat is the most effective, as it inhibits both variants at a lower concentration (12.5 μM), has a low IC_50_ (6.26 μM), and is noncytotoxic at this concentration. Meanwhile, P‐K2, designed to target the spike protein stem, exhibits greater inhibition against the Omicron BA.5.1 variant than against Wuhan D614G and is also noncytotoxic. The ACE2‐mimetic peptides demonstrated their ability to block viral entry by preventing viral‐cell interaction, while the HR2‐mimetic peptide effectively inhibited membrane fusion between the viral and host cells. Both types of peptides contribute to preventing viral replication in mammalian cell lines, highlighting their potential as an antiviral therapy. However, further studies are necessary to confirm their efficacy in animal infection models. We propose that these peptides could be used as prophylactic agents prior to viral infection, as they are specifically designed to block viral entry into host cells.

## Author Contributions

Study concept and design: Ángel G. Alpuche‐Solís, Melvin E. Zúñiga‐Hernández, and Karen L. Reyes‐Barrera. Peptides design: Karen L. Reyes‐Barrera and Edgar D. Páez‐Pérez. Acquisition of data: Melvin E. Zúñiga‐Hernández. Analysis and interpretation of data: All authors. Drafting of the manuscript: Melvin E. Zúñiga‐Hernández. Molecular docking analysis: Sergio N. Hidalgo‐Figueroa and Melvin E. Zúñiga‐Hernández. Critical revision and edition of the manuscript: All authors. Obtained funding: Ángel G. Alpuche‐Solís and Clara I. Espitia Pinzón.

## Ethics Statement

A biosafety protocol for working with SARS‐CoV‐2 in the BSL3 laboratory was approved by the Institutional Biosafety Committee of the UNAM.

## Conflicts of Interest

The authors declare no conflicts of interest.

## Supporting information


Supporting File 1

Supporting File 2

Supporting File 3

Supporting File 4

## Data Availability

The data that support the findings of this study are available on request from the corresponding author. The data are not publicly available due to privacy or ethical restrictions.
