# Supplementary figures and images for: ACE2‐ and HR2‐Mimetic Peptides Inhibit Replication of Two SARS‐CoV‐2 Variants
Source: J Med Virol. 2026 Jul 20;98(7):e71060. doi: 10.1002/jmv.71060 (PMC13382212; doi:10.1002/jmv.71060)

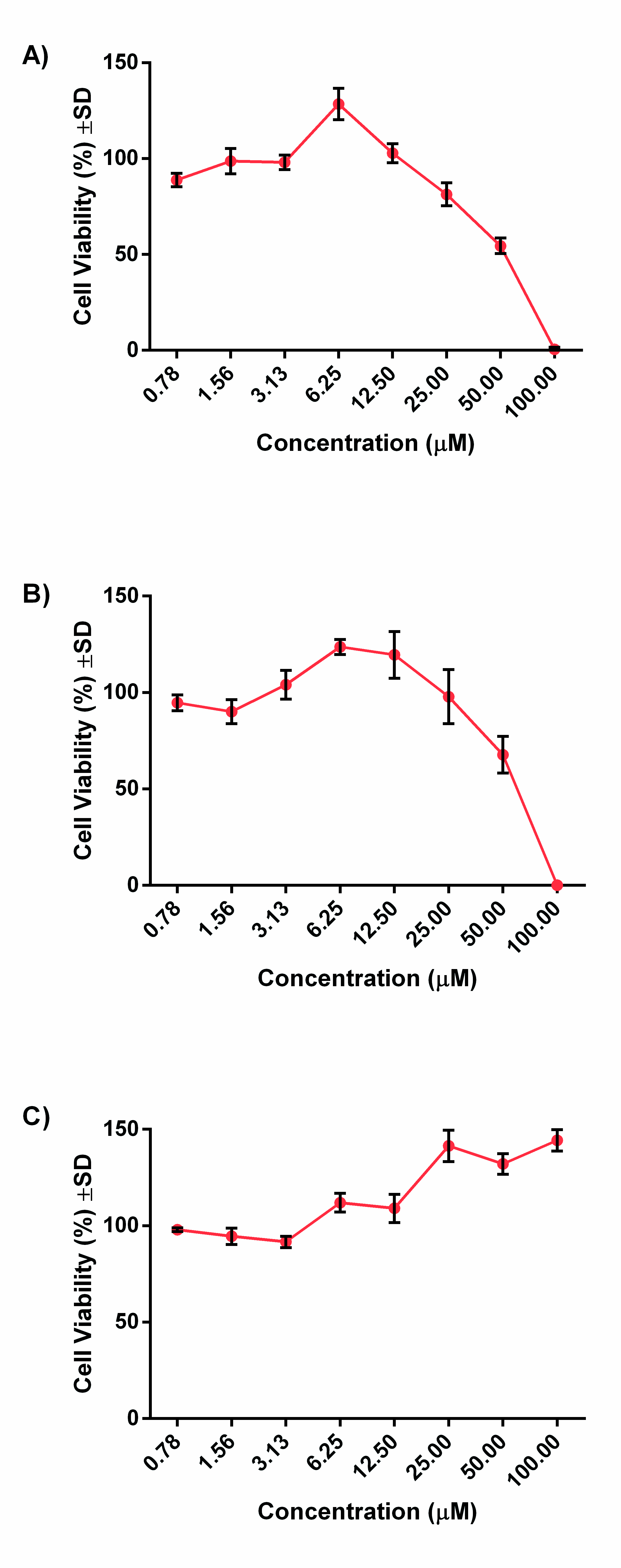

Supplement: Supplementary file 2 — Supporting File 2 [file JMV-98-e71060-s002.tif]
