# Supplementary material for: ACE2‐ and HR2‐Mimetic Peptides Inhibit Replication of Two SARS‐CoV‐2 Variants
Source: J Med Virol. 2026 Jul 20;98(7):e71060. doi: 10.1002/jmv.71060 (PMC13382212; doi:10.1002/jmv.71060)
Supplement: Supplementary file 3 — Supporting File 3 [file JMV-98-e71060-s004.docx]

**Figure S1.** Models of P-Nat, P-3, and P-K2. The models of P-Nat (A) and P-3 (C) were generated using RoseTTAFold, while the model of P-K2 (E) was constructed using Chimera software. **Plots:** The estimated error per amino acid position in the sequences of P-Nat (B) and P-3 (D) is shown, with confidence levels of 0.82 and 0.87, respectively. The plots were generated using RoseTTAFold, which employs a three-track neural network architecture for protein structure prediction.

**Figure S2.** Percentage of cell viability of different peptides. P-Nat (A), P-3 (B), and P-K2 (C) were evaluated in Vero E6 cells for 96 hours. P-Nat and P-3 were cytotoxic at the highest concentrations, while P-K2 was not cytotoxic at any of the concentrations tested.
