# Supplementary material for: ACE2‐ and HR2‐Mimetic Peptides Inhibit Replication of Two SARS‐CoV‐2 Variants
Source: J Med Virol. 2026 Jul 20;98(7):e71060. doi: 10.1002/jmv.71060 (PMC13382212; doi:10.1002/jmv.71060)
Supplement: Supplementary file 4 — Supporting File 4 [file JMV-98-e71060-s001.docx]

**Table S1.** Interaction between P-Nat and RBD of Wuhan. The total energy interaction is -50kcal/mol. A:RBD, E:P-Nat.

| Interaction between amino acids | Parent | Distance | Category | Types | From | From Chemistry | To | To Chemistry |
| --- | --- | --- | --- | --- | --- | --- | --- | --- |
| E:ARG408:HH21 - A:SER23:O | Ligand Non-bond Monitor | 2.93065 | Hydrogen Bond | Conventional Hydrogen Bond | E:ARG408:HH21 | H-Donor | A:SER23:O | H-Acceptor |
| E:TYR473:HH - A:ASP9:OD1 | Ligand Non-bond Monitor | 2.13043 | Hydrogen Bond | Conventional Hydrogen Bond | E:TYR473:HH | H-Donor | A:ASP9:OD1 | H-Acceptor |
| E:SER477:HN - A:GLU2:OE2 | Ligand Non-bond Monitor | 2.856 | Hydrogen Bond | Conventional Hydrogen Bond | E:SER477:HN | H-Donor | A:GLU2:OE2 | H-Acceptor |
| E:GLY502:HN - A:ASP29:O | Ligand Non-bond Monitor | 2.13412 | Hydrogen Bond | Conventional Hydrogen Bond | E:GLY502:HN | H-Donor | A:ASP29:O | H-Acceptor |
| E:TYR505:HH - A:GLY26:O | Ligand Non-bond Monitor | 2.21878 | Hydrogen Bond | Conventional Hydrogen Bond | E:TYR505:HH | H-Donor | A:GLY26:O | H-Acceptor |
| A:GLY26:HN - E:ASP405:OD2 | Ligand Non-bond Monitor | 2.41701 | Hydrogen Bond | Conventional Hydrogen Bond | A:GLY26:HN | H-Donor | E:ASP405:OD2 | H-Acceptor |
| E:ARG408:HD1 - A:SER23:OG | Ligand Non-bond Monitor | 2.84713 | Hydrogen Bond | Carbon Hydrogen Bond | E:ARG408:HD1 | H-Donor | A:SER23:OG | H-Acceptor |
| E:ALA475:HA - A:ASP9:OD2 | Ligand Non-bond Monitor | 2.94802 | Hydrogen Bond | Carbon Hydrogen Bond | E:ALA475:HA | H-Donor | A:ASP9:OD2 | H-Acceptor |
| E:GLY502:HA2 - A:ASP29:O | Ligand Non-bond Monitor | 3.03075 | Hydrogen Bond | Carbon Hydrogen Bond | E:GLY502:HA2 | H-Donor | A:ASP29:O | H-Acceptor |
| A:LYS5:HE1 - E:TYR473:OH | Ligand Non-bond Monitor | 2.7824 | Hydrogen Bond | Carbon Hydrogen Bond | A:LYS5:HE1 | H-Donor | E:TYR473:OH | H-Acceptor |
| A:LYS5:HE2 - E:GLN474:O | Ligand Non-bond Monitor | 2.83404 | Hydrogen Bond | Carbon Hydrogen Bond | A:LYS5:HE2 | H-Donor | E:GLN474:O | H-Acceptor |
| A:GLY26:HA2 - E:ASP405:OD2 | Ligand Non-bond Monitor | 2.82593 | Hydrogen Bond | Carbon Hydrogen Bond | A:GLY26:HA2 | H-Donor | E:ASP405:OD2 | H-Acceptor |
| E:ARG403:NH1 - A:TYR20 | Ligand Non-bond Monitor | 4.10725 | Electrostatic | Pi-Cation | E:ARG403:NH1 | Positive | A:TYR20 | Pi-Orbitals |
| E:ARG408:NH1 - A:PHE19 | Ligand Non-bond Monitor | 4.96406 | Electrostatic | Pi-Cation | E:ARG408:NH1 | Positive | A:PHE19 | Pi-Orbitals |

**Table S2.** Interaction between P-3 and RBD of Wuhan. The total energy interaction is -46.33kcal/mol. A:RBD, E:P-3.

| Interaction between amino acids | Parent | Distance | Category | Types | From | From Chemistry | To | To Chemistry |
| --- | --- | --- | --- | --- | --- | --- | --- | --- |
| E:CYS488:HN - A:GLU16:OE2 | Ligand Non-bond Monitor | 2.18414 | Hydrogen Bond | Conventional Hydrogen Bond | E:CYS488:HN | H-Donor | A:GLU16:OE2 | H-Acceptor |
| A:TYR20:HN - E:GLU484:OE2 | Ligand Non-bond Monitor | 2.3516 | Hydrogen Bond | Conventional Hydrogen Bond | A:TYR20:HN | H-Donor | E:GLU484:OE2 | H-Acceptor |
| A:SER23:HG - E:GLN493:OE1 | Ligand Non-bond Monitor | 2.69965 | Hydrogen Bond | Conventional Hydrogen Bond | A:SER23:HG | H-Donor | E:GLN493:OE1 | H-Acceptor |
| A:GLY29:HN - E:GLY446:O | Ligand Non-bond Monitor | 2.44708 | Hydrogen Bond | Conventional Hydrogen Bond | A:GLY29:HN | H-Donor | E:GLY446:O | H-Acceptor |
| A:PHE31:HN - E:GLY446:O | Ligand Non-bond Monitor | 2.5318 | Hydrogen Bond | Conventional Hydrogen Bond | A:PHE31:HN | H-Donor | E:GLY446:O | H-Acceptor |
| E:GLN493:HA - A:SER23:OG | Ligand Non-bond Monitor | 2.34912 | Hydrogen Bond | Carbon Hydrogen Bond | E:GLN493:HA | H-Donor | A:SER23:OG | H-Acceptor |
| A:GLU16:HA - E:GLU484:OE1 | Ligand Non-bond Monitor | 2.58462 | Hydrogen Bond | Carbon Hydrogen Bond | A:GLU16:HA | H-Donor | E:GLU484:OE1 | H-Acceptor |
| A:PHE19:HA - E:GLN493:OE1 | Ligand Non-bond Monitor | 1.89525 | Hydrogen Bond | Carbon Hydrogen Bond | A:PHE19:HA | H-Donor | E:GLN493:OE1 | H-Acceptor |
| A:SER22:HB1 - E:GLN493:OE1 | Ligand Non-bond Monitor | 2.55194 | Hydrogen Bond | Carbon Hydrogen Bond | A:SER22:HB1 | H-Donor | E:GLN493:OE1 | H-Acceptor |
| A:GLY25:HA2 - E:TYR449:O | Ligand Non-bond Monitor | 2.84032 | Hydrogen Bond | Carbon Hydrogen Bond | A:GLY25:HA2 | H-Donor | E:TYR449:O | H-Acceptor |
| A:GLY29:HA1 - E:GLY446:O | Ligand Non-bond Monitor | 2.88568 | Hydrogen Bond | Carbon Hydrogen Bond | A:GLY29:HA1 | H-Donor | E:GLY446:O | H-Acceptor |
| A:GLU16:OE2 - E:TYR489 | Ligand Non-bond Monitor | 4.91431 | Electrostatic | Pi-Anion | A:GLU16:OE2 | Negative | E:TYR489 | Pi-Orbitals |
| E:PHE490 - A:TYR20 | Ligand Non-bond Monitor | 5.18041 | Hydrophobic | Pi-Pi T-shaped | E:PHE490 | Pi-Orbitals | A:TYR20 | Pi-Orbitals |
| E:TYR449 - A:LYS28 | Ligand Non-bond Monitor | 5.49092 | Hydrophobic | Pi-Alkyl | E:TYR449 | Pi-Orbitals | A:LYS28 | Alkyl |

**Table S3.** Interaction between P-Nat and RBD of Omicron. The total energy interaction is -47.76 kcal/mol. A:RBD, E:P-Nat.

| Interaction between amino acids | Parent | Distance | Category | Types | From |  | From Chemistry | To | To Chemistry |
| --- | --- | --- | --- | --- | --- | --- | --- | --- | --- |
| A:ARG400:HH12 - B:PHE19:O | Ligand Non-bond Monitor | 2.05124 | Hydrogen Bond | Conventional Hydrogen Bond | A:ARG400:HH12 |  | H-Donor | B:PHE19:O | H-Acceptor |
| A:ARG400:HH21 - B:TYR20:O | Ligand Non-bond Monitor | 3.06405 | Hydrogen Bond | Conventional Hydrogen Bond | A:ARG400:HH21 |  | H-Donor | B:TYR20:O | H-Acceptor |
| A:ARG490:HE - B:ASN12:OD1 | Ligand Non-bond Monitor | 2.46325 | Hydrogen Bond | Conventional Hydrogen Bond | A:ARG490:HE |  | H-Donor | B:ASN12:OD1 | H-Acceptor |
| A:HIE502:HE2 - B:TYR20:O | Ligand Non-bond Monitor | 2.65733 | Hydrogen Bond | Conventional Hydrogen Bond | A:HIE502:HE2 |  | H-Donor | B:TYR20:O | H-Acceptor |
| A:HIE502:HE2 - B:GLY24:O | Ligand Non-bond Monitor | 2.35994 | Hydrogen Bond | Conventional Hydrogen Bond | A:HIE502:HE2 |  | H-Donor | B:GLY24:O | H-Acceptor |
| B:ARG31:HN - A:THR497:O | Ligand Non-bond Monitor | 1.75371 | Hydrogen Bond | Conventional Hydrogen Bond | B:ARG31:HN |  | H-Donor | A:THR497:O | H-Acceptor |
| A:GLY499:HA1 - B:ASP29:O | Ligand Non-bond Monitor | 1.98812 | Hydrogen Bond | Carbon Hydrogen Bond | A:GLY499:HA1 |  | H-Donor | B:ASP29:O | H-Acceptor |
| A:GLY499:HA2 - B:ASP29:O | Ligand Non-bond Monitor | 2.1951 | Hydrogen Bond | Carbon Hydrogen Bond | A:GLY499:HA2 |  | H-Donor | B:ASP29:O | H-Acceptor |
| A:ARG400:NH1 - B:PHE19 | Ligand Non-bond Monitor | 3.10983 | Electrostatic | Pi-Cation | A:ARG400:NH1 |  | Positive | B:PHE19 | Pi-Orbitals |
| A:TYR450 - B:PHE19 | Ligand Non-bond Monitor | 4.46101 | Hydrophobic | Pi-Pi Stacked | A:TYR450 |  | Pi-Orbitals | B:PHE19 | Pi-Orbitals |
| A:TYR486 - B:PHE11 | Ligand Non-bond Monitor | 4.5573 | Hydrophobic | Pi-Pi Stacked | A:TYR486 |  | Pi-Orbitals | B:PHE11 | Pi-Orbitals |
| A:ALA481 - B:LEU8 | Ligand Non-bond Monitor | 4.40394 | Hydrophobic | Alkyl | A:ALA481 |  | Alkyl | B:LEU8 | Alkyl |

**Table S4.** Interaction between P-3 and RBD of Omicron. The total energy interaction is -49.14 kcal/mol. A:RBD, E:P-3.

| Interaction between amino acids | Parent | Distance | Category | Types | From | From Chemistry | To | To Chemistry |
| --- | --- | --- | --- | --- | --- | --- | --- | --- |
| A:ARG490:NH2 - B:GLU16:OE1 | Ligand Non-bond Monitor | 3.99809 | Electrostatic | Attractive Charge | A:ARG490:NH2 | Positive | B:GLU16:OE1 | Negative |
| A:ASN484:HN - B:ASP9:OD2 | Ligand Non-bond Monitor | 1.58837 | Hydrogen Bond | Conventional Hydrogen Bond | A:ASN484:HN | H-Donor | B:ASP9:OD2 | H-Acceptor |
| A:PHE487:HN - B:GLU16:OE2 | Ligand Non-bond Monitor | 2.66055 | Hydrogen Bond | Conventional Hydrogen Bond | A:PHE487:HN | H-Donor | B:GLU16:OE2 | H-Acceptor |
| A:GLY482:HA1 - B:HIS13:NE2 | Ligand Non-bond Monitor | 2.74948 | Hydrogen Bond | Carbon Hydrogen Bond | A:GLY482:HA1 | H-Donor | B:HIS13:NE2 | H-Acceptor |
| A:PHE483:HA - B:ASP9:OD2 | Ligand Non-bond Monitor | 2.3215 | Hydrogen Bond | Carbon Hydrogen Bond | A:PHE483:HA | H-Donor | B:ASP9:OD2 | H-Acceptor |
| B:LYS5:NZ - A:PHE483 | Ligand Non-bond Monitor | 4.92563 | Electrostatic | Pi-Cation | B:LYS5:NZ | Positive | A:PHE483 | Pi-Orbitals |
| A:SER491:HN - B:PHE19 | Ligand Non-bond Monitor | 2.99421 | Hydrogen Bond | Pi-Donor Hydrogen Bond | A:SER491:HN | H-Donor | B:PHE19 | Pi-Orbitals |
| B:SER22:OG - A:TYR446 | Ligand Non-bond Monitor | 2.84032 | Other | Pi-Lone Pair | B:SER22:OG | Lone Pair | A:TYR446 | Pi-Orbitals |
| B:SER22:C,O;SER23:N - A:TYR446 | Ligand Non-bond Monitor | 4.89961 | Hydrophobic | Amide-Pi Stacked | B:SER22:C,O;SER23:N | Amide | A:TYR446 | Pi-Orbitals |
| A:PHE483 - B:LYS5 | Ligand Non-bond Monitor | 4.78466 | Hydrophobic | Pi-Alkyl | A:PHE483 | Pi-Orbitals | B:LYS5 | Alkyl |
| A:PHE483 - B:ILE6 | Ligand Non-bond Monitor | 5.43822 | Hydrophobic | Pi-Alkyl | A:PHE483 | Pi-Orbitals | B:ILE6 | Alkyl |
| B:HIS13 - A:ALA481 | Ligand Non-bond Monitor | 4.25439 | Hydrophobic | Pi-Alkyl | B:HIS13 | Pi-Orbitals | A:ALA481 | Alkyl |
| B:PHE19 - A:ARG490 | Ligand Non-bond Monitor | 5.44346 | Hydrophobic | Pi-Alkyl | B:PHE19 | Pi-Orbitals | A:ARG490 | Alkyl |

**Table S5.** Interaction between P-K2 and RBD of Omicron. The total energy interaction is -92.43 kcal/mol. A: strand of hr1 showing no interaction B: left strand of hr1 C: right strand of hr1 D: PK-2.

| Interaction between amino acids | Parent | Distance | Category | Types | From | From Chemistry | To | To Chemistry |
| --- | --- | --- | --- | --- | --- | --- | --- | --- |
| C:LYS947:HZ2 - D:GLU1182:OE1 | Ligand Non-bond Monitor | 2.81806 | Hydrogen Bond;Electrostatic | Salt Bridge;Attractive Charge | C:LYS947:HZ2 | H-Donor;Positive | D:GLU1182:OE1 | H-Acceptor;Negative |
| C:LYS947:HZ2 - D:GLU1182:OE2 | Ligand Non-bond Monitor | 2.79813 | Hydrogen Bond;Electrostatic | Salt Bridge;Attractive Charge | C:LYS947:HZ2 | H-Donor;Positive | D:GLU1182:OE2 | H-Acceptor;Negative |
| D:ARG1185:HH21 - C:ASP936:OD2 | Ligand Non-bond Monitor | 2.16869 | Hydrogen Bond;Electrostatic | Salt Bridge;Attractive Charge | D:ARG1185:HH21 | H-Donor;Positive | C:ASP936:OD2 | H-Acceptor;Negative |
| B:ASN928:HD22 - D:ILE1198:O | Ligand Non-bond Monitor | 2.4315 | Hydrogen Bond | Conventional Hydrogen Bond | B:ASN928:HD22 | H-Donor | D:ILE1198:O | H-Acceptor |
| B:GLN935:HE21 - D:ILE1190:O | Ligand Non-bond Monitor | 1.41062 | Hydrogen Bond | Conventional Hydrogen Bond | B:GLN935:HE21 | H-Donor | D:ILE1190:O | H-Acceptor |
| B:ASN953:HD22 - D:VAL1177:O | Ligand Non-bond Monitor | 1.81972 | Hydrogen Bond | Conventional Hydrogen Bond | B:ASN953:HD22 | H-Donor | D:VAL1177:O | H-Acceptor |
| B:ASN960:HD22 - D:ALA1174:O | Ligand Non-bond Monitor | 2.07116 | Hydrogen Bond | Conventional Hydrogen Bond | B:ASN960:HD22 | H-Donor | D:ALA1174:O | H-Acceptor |
| C:GLN926:HE21 - D:LEU1197:O | Ligand Non-bond Monitor | 1.84519 | Hydrogen Bond | Conventional Hydrogen Bond | C:GLN926:HE21 | H-Donor | D:LEU1197:O | H-Acceptor |
| C:GLN926:HE22 - D:GLU1195:O | Ligand Non-bond Monitor | 1.98277 | Hydrogen Bond | Conventional Hydrogen Bond | C:GLN926:HE22 | H-Donor | D:GLU1195:O | H-Acceptor |
| C:LYS933:HZ1 - D:ASN1192:OD1 | Ligand Non-bond Monitor | 1.89497 | Hydrogen Bond | Conventional Hydrogen Bond | C:LYS933:HZ1 | H-Donor | D:ASN1192:OD1 | H-Acceptor |
| C:LYS933:HZ2 - D:ASN1192:OD1 | Ligand Non-bond Monitor | 1.89677 | Hydrogen Bond | Conventional Hydrogen Bond | C:LYS933:HZ2 | H-Donor | D:ASN1192:OD1 | H-Acceptor |
| C:ASN955:HD22 - D:SER1175:O | Ligand Non-bond Monitor | 2.50435 | Hydrogen Bond | Conventional Hydrogen Bond | C:ASN955:HD22 | H-Donor | D:SER1175:O | H-Acceptor |
| D:ALA1174:HN - B:ASN960:OD1 | Ligand Non-bond Monitor | 1.44942 | Hydrogen Bond | Conventional Hydrogen Bond | D:ALA1174:HN | H-Donor | B:ASN960:OD1 | H-Acceptor |
| D:SER1175:HG - C:GLN954:OE1 | Ligand Non-bond Monitor | 2.49107 | Hydrogen Bond | Conventional Hydrogen Bond | D:SER1175:HG | H-Donor | C:GLN954:OE1 | H-Acceptor |
| D:SER1175:HN - C:ASN955:OD1 | Ligand Non-bond Monitor | 2.18441 | Hydrogen Bond | Conventional Hydrogen Bond | D:SER1175:HN | H-Donor | C:ASN955:OD1 | H-Acceptor |
| D:VAL1177:HN - B:ASN953:OD1 | Ligand Non-bond Monitor | 1.61389 | Hydrogen Bond | Conventional Hydrogen Bond | D:VAL1177:HN | H-Donor | B:ASN953:OD1 | H-Acceptor |
| D:ILE1179:HN - B:GLN949:OE1 | Ligand Non-bond Monitor | 1.50685 | Hydrogen Bond | Conventional Hydrogen Bond | D:ILE1179:HN | H-Donor | B:GLN949:OE1 | H-Acceptor |
| D:ILE1198:HN - B:ASN928:OD1 | Ligand Non-bond Monitor | 2.34548 | Hydrogen Bond | Conventional Hydrogen Bond | D:ILE1198:HN | H-Donor | B:ASN928:OD1 | H-Acceptor |
| C:LYS933:HE2 - D:ASN1192OD1 | Ligand Non-bond Monitor | 2.46648 | Hydrogen Bond | Carbon Hydrogen Bond | C:LYS933:HE2 | H-Donor | D:ASN1192:OD1 | H-Acceptor |
| D:ASN1173:HA - B:ASN960:OD1 | Ligand Non-bond Monitor | 2.01224 | Hydrogen Bond | Carbon Hydrogen Bond | D:ASN1173:HA | H-Donor | B:ASN960:OD1 | H-Acceptor |
| D:ALA1174:HA - C:ASN955:OD1 | Ligand Non-bond Monitor | 2.67648 | Hydrogen Bond | Carbon Hydrogen Bond | D:ALA1174:HA | H-Donor | C:ASN955:OD1 | H-Acceptor |
| D:GLU1176:HA - B:ASN953:OD1 | Ligand Non-bond Monitor | 2.02794 | Hydrogen Bond | Carbon Hydrogen Bond | D:GLU1176:HA | H-Donor | B:ASN953:OD1 | H-Acceptor |
| D:ASN1178:HA - B:GLN949:OE1 | Ligand Non-bond Monitor | 2.04826 | Hydrogen Bond | Carbon Hydrogen Bond | D:ASN1178:HA | H-Donor | B:GLN949:OE1 | H-Acceptor |
| D:LEU1197:HA - B:ASN928:OD1 | Ligand Non-bond Monitor | 2.90639 | Hydrogen Bond | Carbon Hydrogen Bond | D:LEU1197:HA | H-Donor | B:ASN928:OD1 | H-Acceptor |
| D:LEU1203:HA - C:ASN919:OD1 | Ligand Non-bond Monitor | 1.83379 | Hydrogen Bond | Carbon Hydrogen Bond | D:LEU1203:HA | H-Donor | C:ASN919:OD1 | H-Acceptor |
| B:LYS921 - D:LEU1200 | Ligand Non-bond Monitor | 5.11519 | Hydrophobic | Alkyl | B:LYS921 | Alkyl | D:LEU1200 | Alkyl |
| B:ALA924 - D:ILE1198 | Ligand Non-bond Monitor | 4.18626 | Hydrophobic | Alkyl | B:ALA924 | Alkyl | D:ILE1198 | Alkyl |
| B:ALA924 - D:LEU1200 | Ligand Non-bond Monitor | 4.57821 | Hydrophobic | Alkyl | B:ALA924 | Alkyl | D:LEU1200 | Alkyl |
| B:ILE931 - D:LEU1193 | Ligand Non-bond Monitor | 4.96823 | Hydrophobic | Alkyl | B:ILE931 | Alkyl | D:LEU1193 | Alkyl |
| B:LEU938 - D:LEU1186 | Ligand Non-bond Monitor | 5.09228 | Hydrophobic | Alkyl | B:LEU938 | Alkyl | D:LEU1186 | Alkyl |
| B:LEU938 - D:ILE1190 | Ligand Non-bond Monitor | 4.41578 | Hydrophobic | Alkyl | B:LEU938 | Alkyl | D:ILE1190 | Alkyl |
| B:ALA942 - D:ILE1183 | Ligand Non-bond Monitor | 4.6548 | Hydrophobic | Alkyl | B:ALA942 | Alkyl | D:ILE1183 | Alkyl |
| B:ALA942 - D:LEU1186 | Ligand Non-bond Monitor | 4.77949 | Hydrophobic | Alkyl | B:ALA942 | Alkyl | D:LEU1186 | Alkyl |
| B:LEU945 - D:ILE1179 | Ligand Non-bond Monitor | 5.49455 | Hydrophobic | Alkyl | B:LEU945 | Alkyl | D:ILE1179 | Alkyl |
| B:ALA956 - D:ALA1174 | Ligand Non-bond Monitor | 3.12582 | Hydrophobic | Alkyl | B:ALA956 | Alkyl | D:ALA1174 | Alkyl |
| B:VAL963 - D:ILE1169 | Ligand Non-bond Monitor | 4.59861 | Hydrophobic | Alkyl | B:VAL963 | Alkyl | D:ILE1169 | Alkyl |
| B:VAL963 - D:ILE1172 | Ligand Non-bond Monitor | 4.5353 | Hydrophobic | Alkyl | B:VAL963 | Alkyl | D:ILE1172 | Alkyl |
| B:LEU967 - D:ILE1169 | Ligand Non-bond Monitor | 4.65475 | Hydrophobic | Alkyl | B:LEU967 | Alkyl | D:ILE1169 | Alkyl |
| C:LEU922 - D:ILE1198 | Ligand Non-bond Monitor | 5.08939 | Hydrophobic | Alkyl | C:LEU922 | Alkyl | D:ILE1198 | Alkyl |
| C:ILE923 - D:ILE1198 | Ligand Non-bond Monitor | 4.66242 | Hydrophobic | Alkyl | C:ILE923 | Alkyl | D:ILE1198 | Alkyl |
| C:ALA930 - D:LEU1193 | Ligand Non-bond Monitor | 4.99085 | Hydrophobic | Alkyl | C:ALA930 | Alkyl | D:LEU1193 | Alkyl |
| C:LYS933 - D:VAL1189 | Ligand Non-bond Monitor | 4.43713 | Hydrophobic | Alkyl | C:LYS933 | Alkyl | D:VAL1189 | Alkyl |
| C:LYS933 - D:LEU1193 | Ligand Non-bond Monitor | 4.61009 | Hydrophobic | Alkyl | C:LYS933 | Alkyl | D:LEU1193 | Alkyl |
| C:ALA944 - D:ILE1179 | Ligand Non-bond Monitor | 4.00096 | Hydrophobic | Alkyl | C:ALA944 | Alkyl | D:ILE1179 | Alkyl |
| C:VAL951 - D:VAL1177 | Ligand Non-bond Monitor | 4.24297 | Hydrophobic | Alkyl | C:VAL951 | Alkyl | D:VAL1177 | Alkyl |
| C:ALA958 - D:ILE1172 | Ligand Non-bond Monitor | 5.1289 | Hydrophobic | Alkyl | C:ALA958 | Alkyl | D:ILE1172 | Alkyl |
| C:LEU962 - D:ILE1169 | Ligand Non-bond Monitor | 5.29248 | Hydrophobic | Alkyl | C:LEU962 | Alkyl | D:ILE1169 | Alkyl |
| C:LEU962 - D:ILE1172 | Ligand Non-bond Monitor | 5.22951 | Hydrophobic | Alkyl | C:LEU962 | Alkyl | D:ILE1172 | Alkyl |
| D:ALA1174 - B:LEU959 | Ligand Non-bond Monitor | 4.91032 | Hydrophobic | Alkyl | D:ALA1174 | Alkyl | B:LEU959 | Alkyl |
| D:VAL1177 - C:LEU948 | Ligand Non-bond Monitor | 5.30601 | Hydrophobic | Alkyl | D:VAL1177 | Alkyl | C:LEU948 | Alkyl |
